# 66. Utilizing ceftazidime/avibactam therapeutic drug monitoring in the treatment of neurosurgical meningitis caused by Difficult-to-treat resistant (DTR)-*Pseudomonas aeruginosa* and KPC-producing *Enterobacterales*

**DOI:** 10.1093/ofid/ofab466.066

**Published:** 2021-12-04

**Authors:** Mohamad Yasmin, Amir nutman, Lu Wang, Steven Marshall, Dafna Yahav, Ke Chen, Jiping Wang, Jian Li, Robert A Bonomo

**Affiliations:** 1 Case Western Reserve University, Cleveland, Ohio; 2 Beilinson Hospital, Tel aviv, Tel Aviv, Israel; 3 Monash University, Melbourne, Victoria, Australia; 4 Louis Stokes Cleveland Medical Center, Cleveland, OH; 5 Tel Aviv University, Rabin Medical Center; 6 Louis Stokes Cleveland VA Medical Center, Cleveland, OH

## Abstract

**Background:**

Central nervous system (CNS) infections caused by carbapenem-resistant Enterobacterales (CRE) and Difficult-to-treat resistant (DTR)-*Pseudomonas aeruginosa* (PA) are a therapeutic challenge. Data demonstrating the pharmacokinetic/pharmacodynamic (PK/PD) properties of newer beta-lactamase inhibitors remains scarce. A clinical challenge lies in selecting an antimicrobial regimen that diffuses across the blood brain barrier and maintains concentrations to achieve PD targets associated with bacterial killing. These complexities compelled us to quantify the pharmacological properties of ceftazidime/avibactam (CZA). Utilizing therapeutic drug monitoring (TDM), we evaluated the adequacy of therapy and aimed to guide precise CNS dosing in the treatment of three patients with neurosurgical meningitis.

**Methods:**

Bacterial identification and susceptibility testing were performed using MicroScan. TDM of CZA was implemented using a dose of 2.5 g infused intravenously over 2-hours, every 8 hours. The concentrations of ceftazidime and avibactam were determined by liquid chromatography/mass spectrometry. For patients 2 and 3, four unique CSF and plasma samples spanning the dosing interval were obtained; including trough values. (See table)

**Results:**

Bacterial identification and CZA MICs for patients 1, 2, and 3 revealed *bla*_KPC_*Kp* (0.25μg/mL), DTR PA (4 μg/mL), and bla_KPC_*E. cloacae* (0.25 μg/mL), respectively. Measured plasma and CSF concentrations of avibactam (AVI) and ceftazidime (CAZ) are shown in Table 1.

Table 1a. Therapeutic Drug Monitoring of CAZ-AVI depicting dosing, time of samples, and measured concentrations in CSF and Human Plasma (HP)

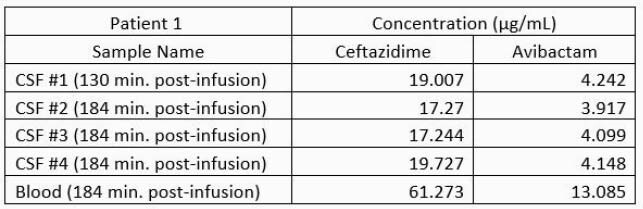

Table 1b. Therapeutic Drug Monitoring of CAZ-AVI concentrations in CSF and Human Plasma (HP) pertaining to patient 2 and 3

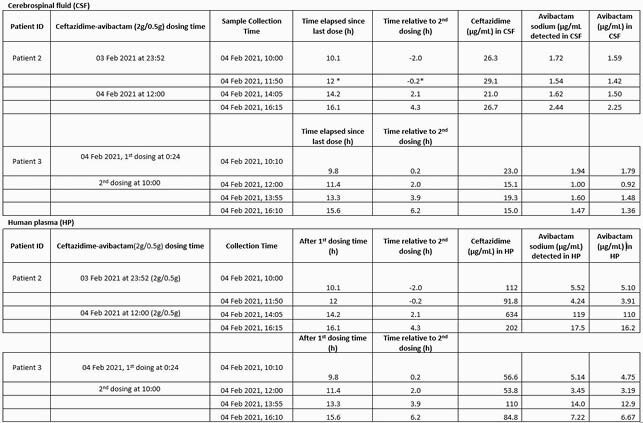

**Conclusion:**

Measuring CZA concentration levels in CSF was achieved in 3 patients with complicated CNS infections. Post-infusion concentrations indicated that adequate CAZ and AVI exposures were attained in the CSF. Notably, avibactam was shown to achieve concentrations ≥1 µg/ml in the CSF throughout the dosing interval. For avibactam and ceftazidime, the PK/PD target correlated with bacterial killing is ~50% fT >MIC. In 2 out of 3 patients, concentrations were determined to be above the respective MICs throughout the entire dosing interval in the CSF. All patients attained clinical and microbiological cure. A novel CZA TDM method was successfully employed to establish that CZA maintains therapeutic CSF concentrations that exceed the MIC throughout the dosing interval.

**Disclosures:**

**Robert A. Bonomo, MD**, **entasis** (Research Grant or Support)**Merck** (Grant/Research Support)**NIH** (Grant/Research Support)**VA Merit Award** (Grant/Research Support)**VenatoRx** (Grant/Research Support)

